# One-Step Grown Carbonaceous Germanium Nanowires and Their Application as Highly Efficient Lithium-Ion Battery Anodes

**DOI:** 10.1021/acsaem.1c03404

**Published:** 2022-01-19

**Authors:** Adrià Garcia, Subhajit Biswas, David McNulty, Ahin Roy, Sreyan Raha, Sigita Trabesinger, Valeria Nicolosi, Achintya Singha, Justin D. Holmes

**Affiliations:** † School of Chemistry & Tyndall National Institute, University College Cork, Cork T12 YN60, Ireland; ‡ AMBER Centre, Environmental Research Institute, University College Cork, Cork T23 XE10, Ireland; § Battery Electrodes and Cells, Electrochemistry Laboratory, Paul Scherrer Institute, Forschungsstrasse 111, 5232 Villigen PSI, Switzerland; ∥ Bernal Institute & Chemical Sciences Department, University of Limerick, Limerick V94 T9PX, Ireland; ⊥ School of Chemistry and CRANN, AMBER Centre, Trinity College Dublin, Dublin 2, Ireland; # Department of Physics, Bose Institute, 93/1, A.P.C. Road, Kolkata 700009, India

**Keywords:** nanowire, germanium, self-seeded growth, supercritical fluid, Li-ion battery

## Abstract

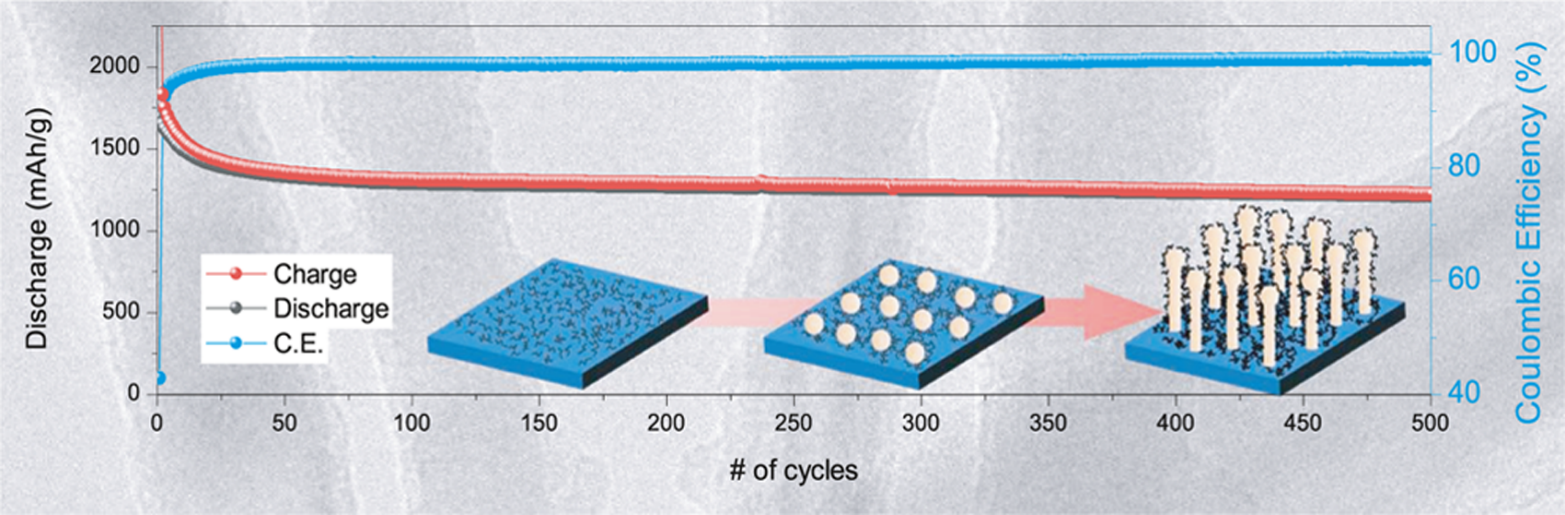

Developing a simple, cheap, and scalable synthetic method for the fabrication of functional nanomaterials is crucial. Carbon-based nanowire nanocomposites could play a key role in integrating group IV semiconducting nanomaterials as anodes into Li-ion batteries. Here, we report a very simple, one-pot solvothermal-like growth of carbonaceous germanium (C-Ge) nanowires in a supercritical solvent. C-Ge nanowires are grown just by heating (380–490 °C) a commercially sourced Ge precursor, diphenylgermane (DPG), in supercritical toluene, without any external catalysts or surfactants. The self-seeded nanowires are highly crystalline and very thin, with an average diameter between 11 and 19 nm. The amorphous carbonaceous layer coating on Ge nanowires is formed from the polymerization and condensation of light carbon compounds generated from the decomposition of DPG during the growth process. These carbonaceous Ge nanowires demonstrate impressive electrochemical performance as an anode material for Li-ion batteries with high specific charge values (>1200 mAh g^–1^ after 500 cycles), greater than most of the previously reported for other “binder-free” Ge nanowire anode materials, and exceptionally stable capacity retention. The high specific charge values and impressively stable capacity are due to the unique morphology and composition of the nanowires.

## 1 Introduction

Group IV materials, especially silicon (Si) and germanium (Ge), continue to gather attention as soon-to-be replacements for graphite as negative electrode materials in energy storage, especially lithium-ion batteries (LIBs), due to the limitations of the traditional carbon-based electrode materials to meet growing demands.^1,2^ Group IV semiconductors, particularly Ge, could be a promising alternative to conventional graphite electrodes, especially in niche energy storage applications like small high-tech devices (such as solar cells^3^ and nanoscale thermoelectric^4^ or electric vehicles^5^), as Ge has a higher theoretical reversible specific charge (1600 mAh g^–1^ for the alloy Li_22_Ge_5_)^6^ than graphite. Ge presents several advantages as LIB anode, such as improved performance at a high charge rate,^7,8^ higher lithium-ion diffusivity, which is about 2 orders of magnitude higher than that in Si,^9,10^ and high electrical conductivity.^11^ However the main drawback of implementing Ge (or Si) as Li-ion battery anode is electrode pulverization after prolonged cycling times, and in turn, severe capacity fading.^9^ Thus, the surface composition, morphology, and composition of the active material are critical for ideal electrode design. However, complex and expensive synthesis steps are usually added to the overall process to achieve the desired surface and morphology.

Shaping the anode material into nanowire form offers a unique solution to the electrode pulverization problem. Nanowires can retain their structural integrity while transitioning from crystalline to amorphous phase during lithiation/delithiation. In addition, nanowires also provide a porous and tunneled architecture with a high interfacial area in direct contact with the electrolyte, thus increasing the energy density in LIBs.^12,13^ Hence, the manufacturing of group IV nanowires, including Ge, for use as anode materials in Li-ion batteries has been widely explored.^14−16^ One of the other significant strategies to inhibit pulverization of Ge (or Si) materials during charging/discharging cycles include carbon encapsulation of Ge nanostructures.^17,18^ Designing Ge anode materials by combining a carbon-based porous structure (amorphous carbon, graphene, reduced graphene oxide, etc.) along with the crystalline nanostructures (i.e., carbonaceous nanostructures) is a possible alternative to graphite anodes for LIBs with high energy densities and long cycling lifetimes.^17,19^

The achievement of the full potential of the one-dimensional (1D) Ge or 1D carbonaceous germanium (C-Ge) nanocomposites in energy storage applications requires development toward simpler and scalable synthetic methods to produce a high yield of nanowires at low cost. Different bottom-up paradigms, such as vapor–liquid–solid (VLS),^20^ vapor–solid–solid,^21^ solution–liquid–solid,^22^ etc., are typically preferred for the 1D growth of phase-pure Ge nanostructures via the use of expensive metal or metalloid catalysts, e.g., Au, Ag, AuAg, etc.^23^ Nanowire growth using metal seeds is not only more expensive but also can lead to impurity incorporation from the metallic seeds into the nanowire structure, which influences the mechanical and electrical properties of the material, and potentially the capacity of Li-ion cells.^24^ On the other hand, the self-seeded growth of Ge nanowires to date has typically involved the use of high-boiling-point organic solvents, high reaction temperatures such as 650–1000 °C,^25^ and designed Ge precursors.^26,27^ While supercritical conditions are already used at the industrial scale,^28^ the achievement of self-induced supercritical conditions with no associated demand of high-pressurized gases is a more scalable and environmentally friendly process. Additionally, carbon (or carbonaceous compounds) embedding of Ge nanostructures, to integrate both elements into a single electrode material, involves ex situ encapsulation methods^7,29,30^ requiring multiple steps and might be difficult to scale up. Numerous efforts involving additional surface chemistry, postgrowth calcination, carbon nanotubes, graphite templates, and metal nanoparticles as catalysts have been adopted to create ideal group IV-based carbon-nanowire nanocomposite materials for use as advanced electrodes in LIBs.^29,31,32^

Here, we report a simple yet cutting-edge method to synthesize self-seeded C-Ge nanowires in a batch reaction process. The single-step batch synthesis method does not involve any additional catalysts (metal or metalloid) and templates. This growth method utilizes a supercritical toluene atmosphere for the nanowire growth and encapsulation with the carbon-based matrix. To the best of our knowledge, this work represents the first solution-phase synthesis of self-seeded Ge or C-Ge nanowires in a low-boiling-point solvent (below precursor’s decomposition temperature, 280–340 °C),^20,33^ such as toluene, using a commercially available precursor. The structural and chemical characteristics of the nanowires were thoroughly analyzed to postulate a growth mechanism for the nanowire growth via the solvothermal-like process under a supercritical environment. The electrochemical performance of the C-Ge nanowires was evaluated via long-term galvanostatic cycling. The C-Ge nanowires exhibit impressive electrochemical performance in terms of specific charge and capacity retention, demonstrating a reversible capacity of >1200 mAh g^–1^ after the 500 cycles, close to the theoretical capacity of Ge.

## 2 Results and Discussion

### 2.1 Growth and Morphology of the Ge Nanowires

Carbonaceous Ge (C-Ge) nanowire growth was achieved under a solvothermal-like one-pot growth with supercritical toluene as a solvent without the use of any catalyst or template (see the Supporting Information for a detailed experimental method). Supercritical fluid reaction conditions were obtained at moderate temperatures without the need for external pressurization of the reaction vessel. The supercritical atmosphere provides ideal conditions for fast precursor decomposition and polymer formation, which is crucial for the self-seeded growth of the C-Ge nanowires.

**Figure 1:**
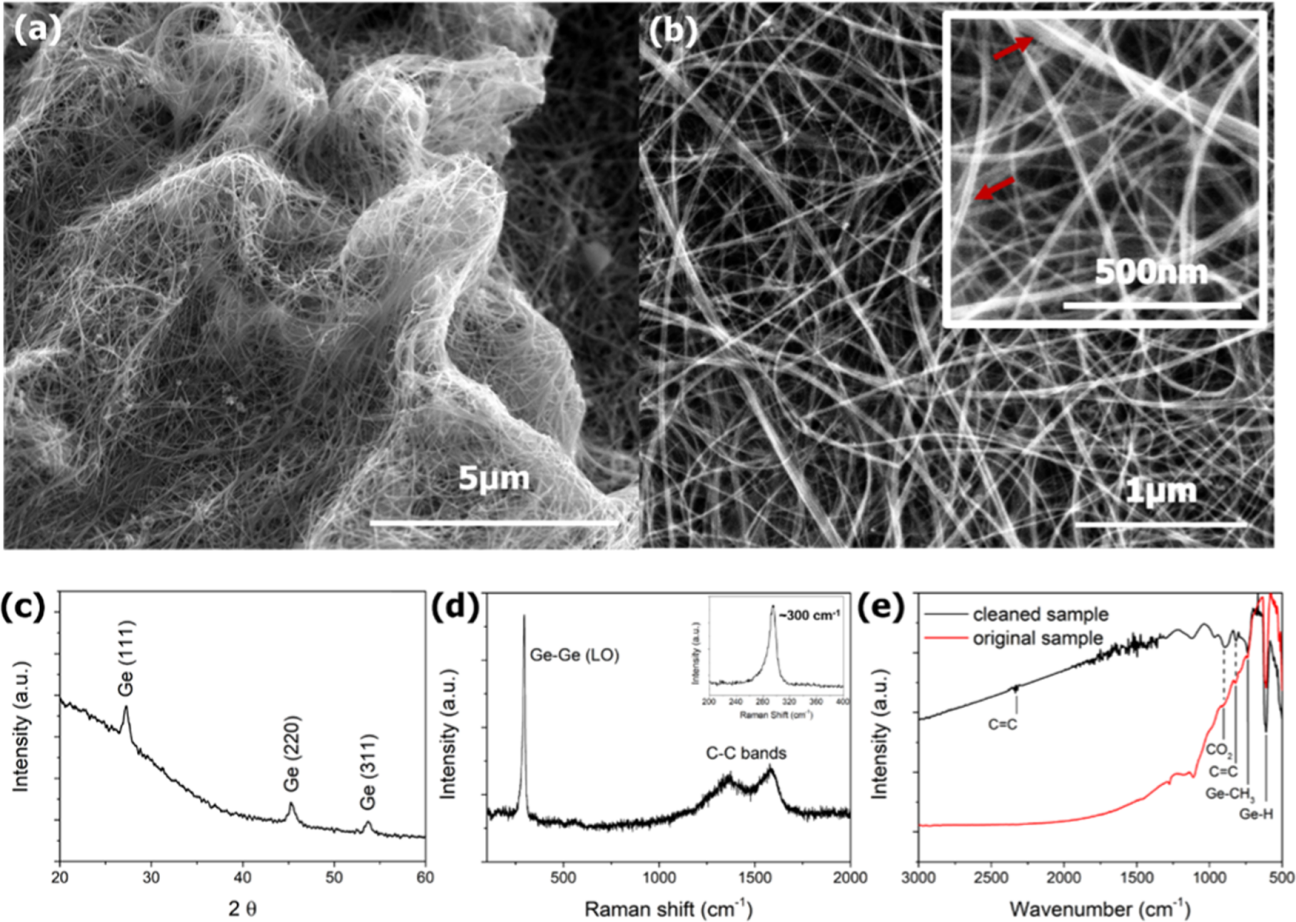
(a, b) SEM micrographs of Ge nanowires grown from a 60 mM DPG/toluene solution at a temperature of 440 °C. The high-magnification SEM image in the inset of (b) highlights the appearance of a core–shell-like morphology in the nanowires (shown by red arrows). (c) X-ray diffraction (XRD) pattern of a nanowire sample showing a diamond cubic structure of Ge crystals. (d) Raman spectrum of C-Ge nanowires displaying two vibrational regions, attributed to Ge–Ge longitudinal optical (LO) (∼300 cm^–1^ peak) and C–C (ca. 1100–1800 cm^–1^) modes. The inset of (d) depicts a detailed view of the Ge–Ge LO area. (e) Fourier transform infrared (FTIR) spectra of the as-grown C-Ge nanowires (red) and the cleaned (with no carbonaceous matrix) Ge nanowires (black) deposited on a Si substrate.

Figure 1a,b shows representative low- and high-magnification scanning electron microscopy (SEM) images of Ge nanowires grown on a Si(100) substrate at a reaction temperature of 440 °C from a 60 mM solution of diphenylgermane (DPG) in toluene. The low-magnification image clearly shows the formation of a three-dimensional porous structure from the interweaving nanowires, which are several micrometers long, consisting of bundles of individual nanowires. The SEM image in Figure 1b and a higher-magnification image shown in the inset show a core/shell-like feature of the nanowires where brightly contrasted nanowire fibers (bundle consisting of several nanowires) are confined in a dark contrasted matrix material (pointed out in the inset of Figure 1b). This dark contrasted matrix can be seen covering all of the nanowires in the sample. This contrast difference may arise due to the different electron conductivity of the crystalline core (Ge) and the surrounding amorphous carbonaceous matrix. SEM analysis also confirmed the presence of negligible amounts of spherical aggregates in samples grown at reaction temperatures of 440 °C. Additionally, no spherical (or hemispherical) growth seeds were detected at the tips of the nanowires, which is typically observed with the catalytic bottom-up nanowire growth. This could be due to the high aspect ratio of the nanowires and the same elemental composition and crystal phase of the seed and nanowire body, which make it difficult to pinpoint the location of the seed at the nanowire tip by SEM observation.

Significantly, the growth of Ge nanowires was also achieved at a very low growth temperature of 380 °C (see Figure S1 in the Supporting Information). SEM analysis showed that nanowires were formed over a range of reaction temperatures and precursor concentrations (see Figure S1 in the Supporting Information). Yields obtained range from 0.18 to 8.74 μg mm^–2^, depending on the growth temperature and precursor concentration. A significant amount of spherical nanoparticle aggregates were observed for the samples synthesized at a higher reaction temperature of 490 °C, due to the homogeneous nucleation and aggregation of Ge growth species (see Figure S1 in the Supporting Information). The presence of these spherical aggregates was negligible at lower growth temperatures (380–440 °C), most likely due to the slow decomposition rate of DPG at these temperatures.

### 2.2 Structural Analysis of Ge Nanowires

X-ray diffraction (XRD) was used to characterize the phase and crystallinity of the nanowires. Figure 1c shows a diffraction pattern from a nanowire sample grown from a 60 mM DPG/toluene solution at a reaction temperature of 380 °C and confirms the formation of crystalline Ge. Reflections at 27.4, 45.4, and 53.8° can be assigned to the (111), (220), and (311) crystallographic planes of the diamond cubic crystal structure of elemental Ge (JCPDS, reference pattern 04-0545), respectively, after subtracting the reflection peaks from the Si substrate. All of the nanowire samples grown had the diamond cubic structure of Ge. No crystallinity was associated with the carbon-based matrix. The presence of the carbon-based coating is further verified through energy-dispersive X-ray (EDX) analysis, transmission electron microscopy (TEM) analysis, X-ray photoelectron spectroscopy (XPS), and Raman spectroscopy.

### 2.3 Chemical Analyses of Ge Nanowires and Carbon-Based Matrix

Raman spectroscopy was used for the qualitative assessment of the Ge nanowires, the carbonaceous matrix and their interaction. Raman vibrations in two well-separated regions were observed and attributed to Ge–Ge degenarate LO-TO mode (∼300 cm^–1^ peak) and C–C (between 1100 and 1800 cm^–1^) modes (representative Raman spectra are shown in Figure 1d). The Raman peak near 300 cm^–1^ was fitted using Lorentzian functions. The sharp Ge–Ge LO peak at 294 cm^–1^ was red-shifted compared to the characteristic value for bulk Ge,^23^ which could be attributed to a combination of effects, such as phonon confinement, due to the narrow diameter of the nanowires (mean diameter ∼13 nm)^34^ and strain caused by the carbon-based matrix.^35^ The observation of C–C Raman modes between 1100 and 1800 cm^–1^ can be assigned to the characteristic feature of the carbon-based coating around the Ge nanowires.^36,37^ A graphitic (G band) at ∼1576 cm^–1^ is associated with the vibrational mode E_2g_ in graphite-like structures, and a disordered band (D band) at ∼1356 cm^–1^ can be assigned to disorder-allowed phonon modes.^38^

The chemical characteristics of the darkly contrasted nanowire matrix, as depicted in the SEM image (see Figure 1b), and its interaction with the Ge nanowires were analyzed by EDX analysis, Fourier transform infrared (FTIR) spectroscopy, and XPS. While EDX analysis shows the presence of carbon on the nanowire (see the dark-field scanning transmission electron microscopy (STEM) image and the corresponding mapping in Figure S2 in the Supporting Information), this is not conclusive for the analysis of surface carbon in nanostructures. The hydrocarbon layers are often built upon materials when subjected to electron beam in the presence of oil vapors from the vacuum system. The representative FTIR spectra from the nanowires showed absorption bands at ∼568, ∼610, and ∼737 cm^–1^ (see Figure 1e). These bands have previously been assigned to the stretching modes of C–Ge–C,^39^ Ge–C^40^ bonds, and wagging mode of Ge–H_3_,^41^^41^ respectively. The FTIR peak at ∼890 cm^–1^ is related to Ge–CH_3_ rocking vibrations.^39,42^ These data confirm the formation of a carbonaceous structure around the crystalline nanowires due to the presence of peaks at ∼963 and ∼815 cm^–1^, corresponding to C=C, as well as ∼890 cm^–1^, which is associated with the presence of CH_3_ structure. XPS analysis was performed (see Figure S3 in the Supporting Information) on representative nanowire samples grown at different reaction temperatures (380 and 440 °C) and with a DPG concentration of 60 mM, to study the oxide formation upon exposure of the samples to air. Germanium oxide formation was found due to air exposure of the samples, when stored in ambient atmosphere after a month (8.4% of GeO and 2.4% of GeO_2_) and a year (8.3% of GeO and 17.4% of GeO_2_). No strong interaction (covalent bonding) between carbon and nanowires surface was found in the C 1s spectrum. However, C–C, C=C, C–O, and CO_3_ bonds were found in the C 1s spectrum. The carbonaceous matrix is most likely porous since it does not act as a passivation layer to protect the Ge nanowires from long-term oxidation. Furthermore, the binary phase diagram of C and Ge shows that the formation of solid solutions of C–Ge is not likely at our growth temperature,^43^ implying that any carbon formed around the Ge nanowires is preferably bound by physisorption. Both XPS and FTIR analyses confirm the presence of carbonaceous materials in the samples and the interaction of the carbon as a physically adsorbed matrix on the surface of the Ge nanowires. XPS, XRD, EDX, and FTIR analyses did not show any impurities in the sample, which could be associated with the nanowire growth and conductive properties of nanowires.

### 2.4 Structural and Crystal Quality Analyses of Nanowires and Carbonaceous Matrix

**Figure 2:**
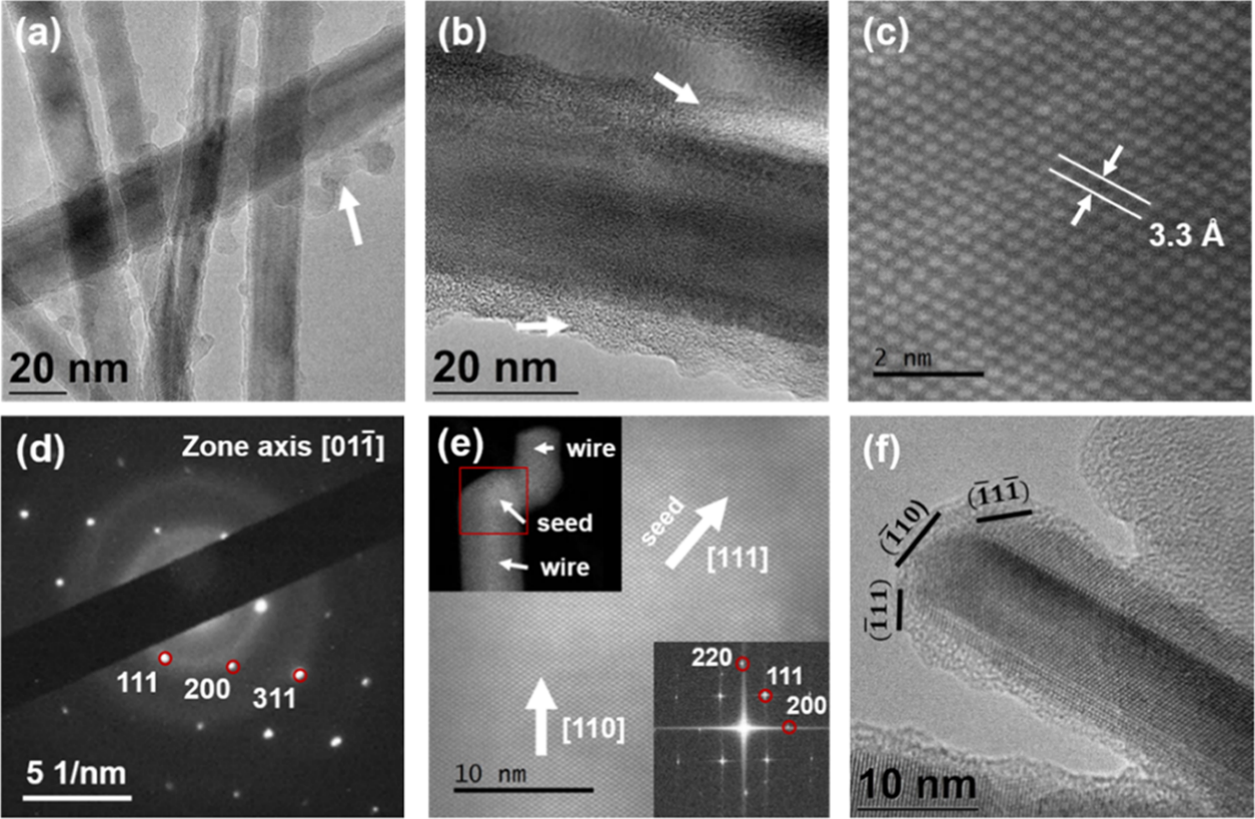
TEM images of C-Ge nanowires showing (a) the discrete deposition of amorphous C-based layer on the Ge nanowires and (b) a continuous deposition of the amorphous shell. Some carbon-matrix areas are highlighted with white arrows. (c, d) Latticed-resolved high-resolution transmission electron microscopy (HRTEM) image and the corresponding selected area electron diffraction (SAED) pattern of a representative Ge nanowire (diamond cubic crystal structure). (e) HRSTEM image of a Ge seed and nanowire interface with a ⟨110⟩ growth direction. The red box on the STEM image in the top-left inset represents the magnified area of the main image. The bottom-right inset shows an fast Fourier transform (FFT) of the full image. The TEM image in (f) shows faceted termination of a Ge nanowire.

To determine the structural quality of the Ge nanowires and the characteristics of the carbon-based shell, C-Ge nanowires were investigated by high-resolution TEM (see Figure 2). Figure 2 shows representative TEM images of nanowires grown at a reaction temperature of 440 °C; nanowires grown at all growth temperatures displayed similar structural quality. Figure 2a,b shows bright-field TEM images of the nanowires. A low-contrast coating on the nanowires is clearly visible in these TEM images. This thick (∼8–10 nm) coating is likely to be of amorphous carbonaceous shell. Such low-contrast carbon-based coatings are present in two different kinds of disposition: (i) as free aggregates between the nanowires (see Figure 2a) and (ii) as an uneven coating along the nanowire length (see Figure 2b). The amount of carbonaceous compound is estimated to be between 5 and 10 wt % of samples content. The amount of carbonaceous compounds was calculated by analyzing the width of the core–shell structure and the dimension of dispersed carbon in the sample. Ge nanowires grown at 440 °C were found to have a mean diameter of 12.2 (±3.0) nm. The mean diameter of the nanowires was found to vary with reaction temperature (see Figure S4 in the Supporting Information), i.e., 11.3 (±2.2) nm at 380 °C and 19.7 (±4.3) nm at 490 °C. Assuming that nanowire growth is self-seeded, higher reaction temperatures provoke a faster precursor decomposition, leading to the nucleation and agglomeration of larger Ge seed nanoparticles, resulting in the growth of larger-diameter nanowires. Most of the nanowires had lengths >4 μm, with very few individual nanowires displaying bending or kinking. Tapered nanowires were also not observed under any growth conditions investigated.

To determine the crystal quality of the nanowires, their structure was investigated by high-resolution transmission electron microscopy (HRTEM), high-angle annular dark-field (HAADF)-STEM, and selected area electron diffraction (SAED) (see Figure 2c–f). HRTEM and HAADF-STEM of Ge nanowires (see Figure 2c and e), revealed their highly crystalline nature, without any crystal defects such as stacking faults and twinning. Defect-free materials allow for long life cycles and are imperative for their use as Li-ion anode materials.^44^ HRTEM from the core of the crystalline Ge nanowires revealed an interplanar spacing (*d*) of 0.33 nm (see Figure 2c), which is marginally larger than the *d* value for bulk diamond Ge crystals of 0.326 nm (JCPDS 04-0545), corresponding to (111) planes of Ge diamond cubic crystal structure. SAED pattern (see Figure 2d) corresponds to cubic germanium, and the spot pattern indicates that the Ge nanowires are single crystalline. SAED showed a pseudohexagonal symmetry, and the reflections can be assigned to the high-order Laue zone diffraction of {111}, {311}, and {200} planes in group IV diamond cubic crystals.

The nanowire growth direction and the seed–nanowire interface were further examined by HR-STEM (see Figure 2e). A low-magnification image of a Ge nanowire with a growth seed can be seen in the inset of Figure 2e (top-left). A high-magnification image recorded with ⟨1̅10⟩ zone axis alignment, from the same nanowire, shows seamless growth of the nanowire from the seed. Notably, many of the nanowires analyzed displayed an onset of growth in two different directions, from a common nanoparticle seed. However, only one of the growth directions prevailed, increasing to microns in length, while the other end terminated within a few nanometers (top-left inset of Figure 2e). The presence of the nanoparticle at the tip of the nanowire points toward a seeded bottom-up mechanism for the onset of the growth of nanowires. Nanowires grow along the ⟨110⟩ direction (also confirmed from fast Fourier transform (FFT) in the inset), as expected for seeded nanowires of this diameter range.^45,46^ The crystal structure in both the “seed” and the nanowire segments correspond to the diamond cubic crystal of Ge. No crystallographically different interface was observed between the “seed” and the nanowire segments. However, different crystallographic orientations between the “seed” and the nanowires are also present, as shown in Figure 2e, where {111} stacking along the growth orientation is observed in the “seed” segment and the planes in the nanowires are stacked along {110} equivalent directions. The apparent continuity of the similar lattice from the seed to the nanowire confirms the self-seeded, i.e., seeded from in situ grown Ge nanoparticles, formation of the nanowires. Most of the nanowires were found to exhibit distinctive faceting (see Figure 2f). This behavior was previously observed by Lieber et al.,^47^ where they described the preferred ⟨110⟩ growth direction in small diameter Si nanowires with a “V-shaped” termination between Au seeds and the wire. The tapered structure consisted of two {111} facets at 55° relative to the [110] direction, which is related to the surface energy of the crystal facets.^47,48^

### 2.5 Growth Mechanism of Ge Nanowires

**Figure 3:**
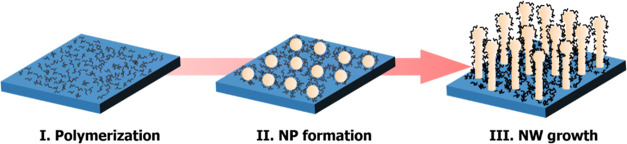
Illustration of the proposed “self-seeded” nanowire growth mechanism. Stage I corresponds to precursor decomposition and polymerization of the liberated phenyl groups. Stage II consists of aggregation and nanoparticle formation from the available Ge adatoms. Eventually, stage III represents nucleation and growth of C-Ge nanowires from the outer disposed Ge nanoparticle seeds.

The growth of the self-seeded nanowires can be explained by a three-phase (source-seed-nanowire) bottom-up growth mechanism. Spontaneous in situ formation of Ge seeds and their participation in the nanowire growth is evident from the presence of Ge nanoparticles at the tip of the nanowire (see Figures 2e and S5b in the Supporting Information) and the formation of Ge nanoparticles in an amorphous matrix (Figure S5a in the Supporting Information). Although classical round-shaped nanoparticles were barely found at the tips, single-crystal faceted structures were always present. The presence of a third-party catalyst seed, such as Au, often acts to enhance precursor decomposition.^22^ However, in this self-catalytic growth process, temperature and pressure are the key factors associated with the initial decomposition of the Ge precursor. Schematic diagrams, SEM and TEM images (see Figures 3 and S5 in the Supporting Information) outline the different stages of nanowire formation via the supercritical batch synthesis. During “stage I” of the growth, the Ge precursor (DPG) decomposes to form Ge adatoms, liberating very reactive phenyl groups.^22^ Gas chromatography-mass spectrometry (GC-MS) analysis (see Figure S6a in the Supporting Information) of the reactant solution revealed the byproducts of the reaction as diphenylmethane and derivatives (e.g., 2,3′-dimethyl-1,1′-biphenyl, bibenzyl or 1-methyl(4-phenylmethyl)benzene) along with toluene (and derivatives), and tetraphenylgermane. Under supercritical condition, phenyl-based long-chain molecules (e.g., diphenylmethane and derivatives) start polymerizing^20^ and precipitate over the available surfaces (sample substrate and reactor’s walls). This is to be noted that self-seeded nanowire growth was not observed for a flow-through reaction under supercritical fluid conditions. Instead, large spherical particles of Ge via homogeneous nucleation and Ostwald ripening were noticed. This could be due to the large localized concentration of diphenylmethane and derivatives in the batch reaction process compared to a flow-through reaction. The formation of tetraphenylgermane is associated with the decomposition of the DPG precursor by phenyl redistribution, as previously reported.^22^

The available Ge adatoms aggregate and dissolve into these polymers to form the Ge nanoparticle seeds, capped by the polymers (stage II of the growth; see Figures 3 and S5a in the Supporting Information). The in situ formed polymeric templates prevent the Ge nanoparticles from large-scale aggregation and Ostwald ripening and thus the formation of larger spherical particles. Eventually, the Ge nanoparticles exposed at the outer surface of the polymer act as a nucleating seed for the growth of Ge nanowires (stage III, see Figures 3 and S5b in the Supporting Information). Self-seeded growth of Ge nanowires has been previously proposed^20,27^ in a “pseudo”-VLS-like growth with the formation of a liquid organic spherical seed in high-boiling-point solvents. These models account for nanoparticle coalescence at the initial stage of nanowire growth, followed by Ostwald ripening in later growth phases.^27^ In our case, the initial formation of Ge nanoparticles in the template of carbonaceous compounds (see Figure S5a in the Supporting Information) and the presence of these nanoparticles with symmetric crystal lattices at the tips of the nanowires (see Figures 2e and S5b in the Supporting Information) suggest the participation of nanoparticle seed in the growth of single-crystal Ge nanowires. Under our growth conditions, Ge nanoparticles with diameters between 11 and 20 nm should remain in a solid state during the nanowire growth process, due to the negligible depression of bulk Ge melting point (∼938 °C) due to the nanoscale effect at these dimensions.^49^ Participation of a solid-state seed is further confirmed by the well-faceted termination of the nanowires (see Figure 2f). During stage III of the growth, the polymeric materials present in the reaction chamber can further precipitate onto the sidewalls of the nanowires to create a carbonaceous coating around the nanowires (see Figure 2a,b). The possibility of nanowire growth in solution, without any substrate, is rejected due to the insignificant quantity of nanowires found in the residual liquid in the reactor after the reaction. Precursor concentration was also not found to play a key role in the quality of the nanowires grown.

Reaction temperatures and loading volumes were the two main variables to influence nanowire growth. The autogenic pressure reached in the reaction system depended on the loading volume of the solvent. Both growth constraints were used to calculate the density of the supercritical phase in the reaction cells during the nanowire growth process (see Figure S6b,c in the Supporting Information). A minimum reaction temperature of 350 °C and a volume fraction of 60% were required to grow carbonaceous Ge nanowires in our setup, with significant nanowire growth achieved at 380 °C (0.35 μm mm^–2^). At a reaction temperature of 350 °C, toluene was in a supercritical state (critical temperature (*T*_c_) = 293.75 °C and critical pressure (*P*_c_) = 598.47 psi, for pure toluene) for only 20 min (see Figure S6b,c), resulting in a low yield of nanowires. A 60% filling of the volume fraction with toluene was also found to be ideal for obtaining homogeneous nanowires with uniform diameters (see Figures S1 and S4 in the Supporting Information). No nanowire growth was achieved with 20% filling volume of the reaction cell with toluene, where no supercritical phase was achieved in the reaction (see Figure S6c in the Supporting Information). A time-dependent evolution of phases of the reaction solution and associated formation of different morphologies are shown in Figures S5 and S6 in the Supporting Information. Stage I of nanowire growth is associated with initial precursor decomposition and nanoparticle formation in the liquid phase (see Figures S5c and S6c in the Supporting Information). Supercritical phase was achieved after a certain interval (different for different loading volumes of the reaction cell and growth temperature); see Figure S6b,c in the Supporting Information. Initial formation of the nanowire could be associated with the initiation of the supercritical phase (Figures S5 and S6c). Further nanowire growth was continued only in the supercritical atmosphere. Thus, it can be concluded that the self-seeded growth of carbonaceous nanowire was only successful when a supercritical phase in the toluene solvent was achieved for a considerable time and a certain range of densities (see Figure S6 in the Supporting Information). Of note, elemental, structural, and chemical analyses did not indicate any presence of metal (or semimetal) impurities that can participate (in a three-phase growth paradigm) in the seedless growth of Ge nanowires. Additionally, solvent and precursor were also sourced from different suppliers (Sigma-Aldrich, ABCR-GmbH, Flurochem, Merck) to negate any influence of impurities on the seedless nanowire growth.

### 2.6 Electrochemical Analysis of C-Ge Nanowires

The electrochemical performance of C-Ge nanowires was evaluated for its potential as anode material in LIBs. To evaluate the electrochemical performance, C-Ge nanowires were grown directly onto Ti foil current collectors. Nanowire growth on the Ti foil was achieved with 40 mM DPG, 60% of loading volume of solvent and at an optimal growth temperature of 440 °C (SEM image in Figure S7 in Supporting Information). As all of the nanowires grown at different growth conditions (i.e., 380 and 440 °C growth temperature, 40 and 60 mM DPG concentrations, 60% loading volume) showed similar morphological and structural properties, electrochemical analysis from this particular sample is representative of all C-Ge nanowires grown with different growth constraints. As previously stated, the estimated amount of carbonaceous compound is between 5 and 10 wt % of samples content, which corresponds to a C/Ge molar ratio of around 1:3. Nanowires directly grown on current collecting substrates do not require initial processing steps, such as decorating the substrate with metal nanoparticle seeds and the use of binders or conductive additives. Additionally, Ti is very inert (unlike Cu foil) as a catalyst for the self-seeded Ge nanowire growth at our growth temperatures, thus keeping the morphological and structural quality of the nanowires similar to that grown on Si substrates (see Figure S7 in the Supporting Information).

**Figure 4:**
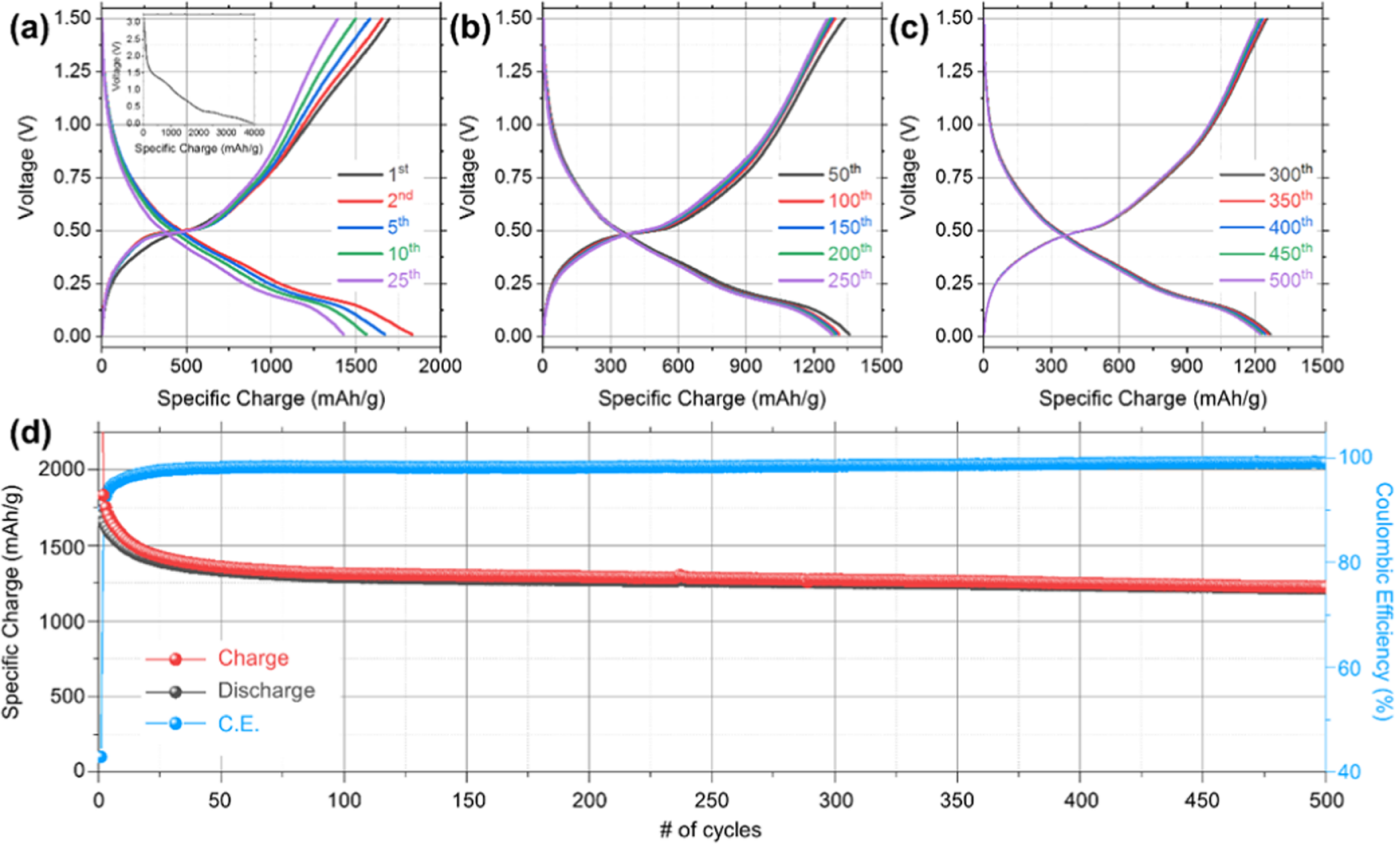
Voltage profiles for (a) the 1st, 2nd, 5th, 10th, and 25th cycles, (b) the 50th, 100th, 150th, 200th, and 250th cycles, and (c) the 300th, 350th, 400th, 450th, and 500th cycles for C-Ge nanowires at 0.2 C in a potential window of 1.50–0.01 V (vs Li/Li^+^). (d) Comparison of the specific charge values and Coulombic efficiency obtained for C-Ge nanowires over 500 cycles.

C-Ge nanowires were cycled galvanostatically for 500 cycles at a rate of 0.2 C, in a voltage range of 1.50–0.01 V (vs Li/Li^+^). A selection of the resulting voltage profiles from the 1st to the 500th cycle is shown in Figure 4a–c. There was an initial rapid decrease from the open-circuit voltage of ∼3.14 V down to ∼0.4 V, during the first charge, as can be seen in the inset of Figure 4a. This sharp voltage decrease may be associated with several processes, including the initial formation of a solid electrolyte interphase (SEI) layer, the irreversible decomposition of the electrolyte on the surface of the electrode material, and the lithiation of crystalline Ge.^50^ Three reduction plateaus can be seen during the initial charge from ∼0.35 to 0.25 V, from 0.25 to 0.15 V, and from 0.15 to 0.01 V, which are attributed to the step-by-step lithiation of the Ge nanowires, leading to the formation of the c-Li_15_Ge_4_ phase.^51^ The oxidation plateau centered at ∼0.49 V during the first discharge is due to the delithiation of the Ge nanowires, and this plateau remains consistently at this potential for the remainder of the 500 cycles.^52^ The initial charge and discharge specific charge values were ∼3947 and 1692 mAh g^–1^, respectively, corresponding to an initial Coulombic efficiency (ICE) of 42.9%. The large initial charge capacity is likely due to the formation of an SEI layer on the surface of the nanowires as well as the initial lithiation of C-Ge, and the presence of high-surface-area amorphous carbon.^53^ The ICE value obtained for our C-Ge nanowires is comparable to previously reported values for other Ge nanowire anodes.^54,55^ Low ICE is a common issue for alloying mode anode materials such as Ge and Si; however, there are some reports that indicate that prelithiation of the anode material can improve the ICE. For example, Si carbon via mechanical pressing of stabilized lithium metal powder onto the working electrode, leading to an increase in ICE values.^56^ Furthermore, the amorphous carbon coating may be contributing to the low ICE. The additional surface area provided by the carbon coating may contribute to the high inefficiency of the first cycle. A future work of interest could be the additional treatment of the C-Ge nanowires, such as heating to higher temperatures that may result in a more graphitized crystalline carbon, which could increase the efficiency of the first few cycles. Three sloping reduction plateaus were observed from the second charge onward from ∼0.70 to 0.45 V, from 0.45 to 0.3 V, and from 0.3 to 0.01 V, which are associated with the formation of a series of Li–Ge alloys (a-Li*_x_*Ge → a-Li_15_Ge_4_ → c-Li_15_Ge_4_).^57^ The presence of these reduction plateaus in the charge curves from the 2nd to the 500th cycle, as well as the consistency of the oxidation plateau, indicates that the formation of the various Li–Ge alloys is a highly reversible process.

The specific charge values obtained for C-Ge nanowires cycled at 0.2 C over 500 cycles and their related Coulombic efficiency (CE) values are shown in Figure 4d. It is clear that large specific charge values and excellent capacity retention can be obtained for C-Ge nanowires grown directly on Ti foil. The specific charge after the 2nd charge was ∼1831 mAh g^–1^, and it decreases to 1376 mAh g^–1^ after the 40th cycle. The CE from the 40th cycle onward is >98% and continued above this value for the remainder of the 500 cycles (see Figure 4d). This level of CE stability is notable for Ge nanowires directly grown on a current collecting substrate in the absence of binders and conductive additives. The specific charge retention from the 40th charge, once the CE stabilized above 98%, to the 500th charge was ∼89%. This is an impressive level of specific charge retention over long-term cycling. The mean capacity decay from the 2nd to the 500th cycle was ∼1.2 mAh g^–1^ per cycle, which is a further indicator of the noteworthy stable cycling observed for the Ge nanowires.

**Figure 5:**
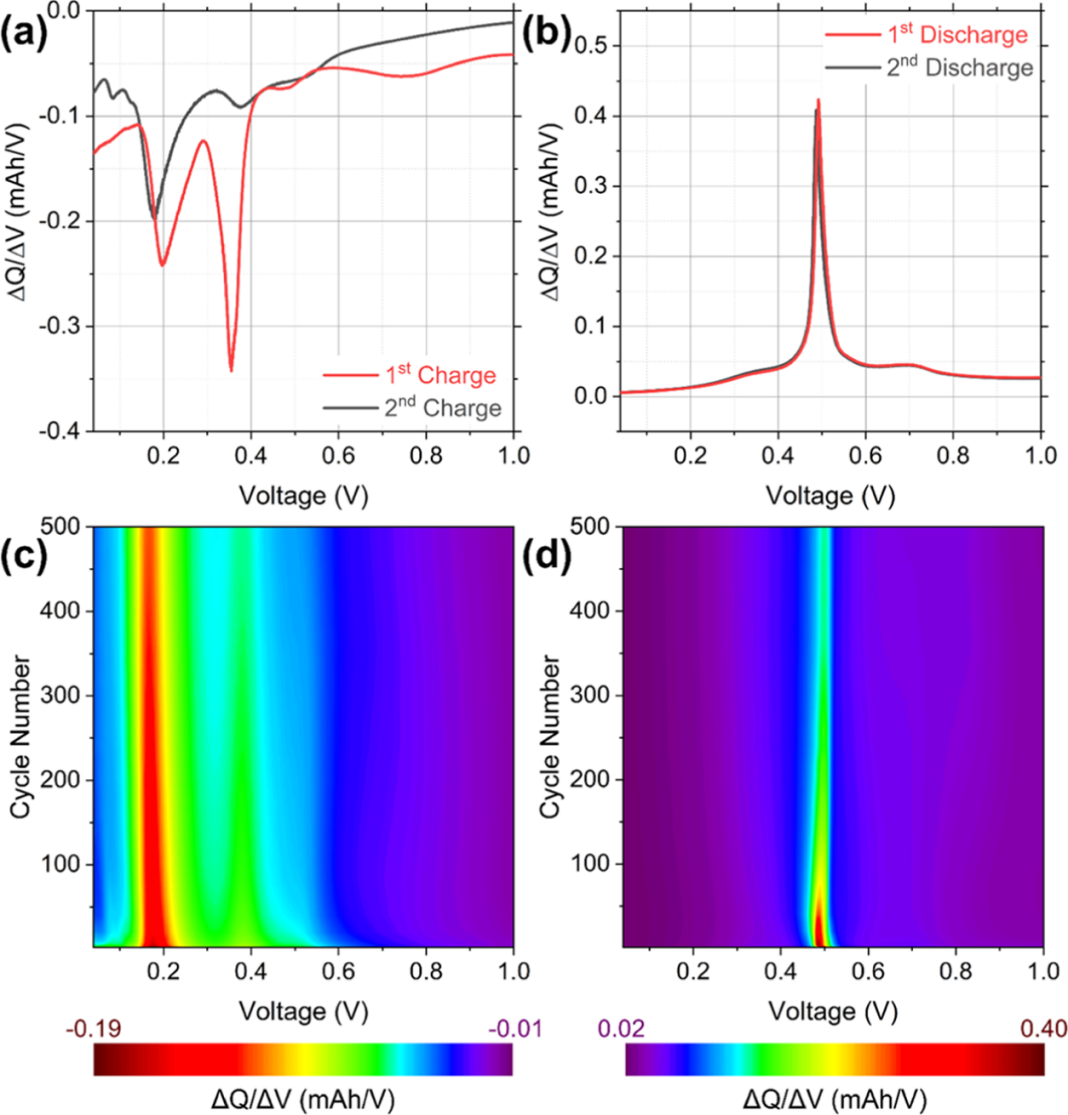
(a) Differential capacity curves calculated from the first and second galvanostatic charges at 0.2 C. (b) Differential capacity contour plot calculated from differential charge curves from the 2nd to the 500th charge. (c) Differential capacity curves calculated from the first and second galvanostatic discharges at 0.2 C. (d) Differential capacity contour plot calculated from differential discharge curves from the 2nd to the 500th discharge.

Differential capacity plots (DCPs) were calculated from galvanostatic charge and discharge curves to investigate the charge storage mechanism of the C-Ge nanowires in more detail. The DCP from the first charge curve consisted of a series of reduction peaks as shown in Figure 5a. The weak band at ∼0.76 V may be associated with the formation of an SEI layer as it is only observed during the first cycle.^19,58^ The strong, sharp peak at ∼0.35 V is associated with the initial lithiation of crystalline Ge, and the intensity of this peak decreases significantly after the first charge. This indicates that the nanowires likely do not return to a fully delithiated, crystalline Ge phase after the initial lithiation of the nanowires. A similar decrease of this reduction peak after the first cycle has previously been reported for other Ge nanowire anodes.^19,23^ The wide asymmetric reduction peak at ∼0.20 V is associated with the formation of a-Li_15_Ge_4_ and c-Li_15_Ge_4_ phases.^59^ From the second charge onward reduction peaks were observed at ∼0.53, 0.38, and 0.18 V, corresponding to the formation of a-Li*_x_*Ge, a-Li_15_Ge_4_, and c-Li_15_Ge_4_ phases, respectively.^50,57^ The strong oxidation peak observed in the DCP of the first discharge, centered at ∼0.49 V, corresponds to the overlapping delithiation of the c-Li_15_Ge_4_ and a-Li_15_Ge_4_ phases.^10,60^ There was no significant shifting of this peak in the DCP from the second discharge. Differential capacity contour plots, calculated from charge and discharge voltage profiles, ranging from the 2nd to the 500th cycles are shown in Figure 5c,d. The two high-intensity regions observed in Figure 5c are associated with the reduction peaks for the formation of the a-Li_15_Ge_4_ and c-Li_15_Ge_4_ phases, which are centered at ∼0.38 and 0.18 V, respectively. The formation of these alloys is a highly reversible process, as the potential of these reduction peaks remains consistent throughout the 500 cycles. Likewise, the oxidation peak at ∼0.49 V remains stable over 500 cycles as shown in Figure 5d. The consistency of these reduction and oxidation peaks may give rise to the stable capacity retention observed in Figure 4d.

Deformation and electrochemical restructuring of nanowire morphology and amorphization of the Ge-C nanomaterial, similar to Ge nanowires,^59^ was observed after 500 cycles (Figure S8 in the Supporting Information). The formation of a mesh of active material by agglomeration of individual nanowires was previously observed for phase-pure Ge nanowire after 100 cycles.^59^ However, compared to the phase-pure Ge nanowires Ge-C nanowires show better retention of nanowire morphology with the withholding of the cylindrical shape for many nanowires after 500 cycles (Figure S8 in the Supporting Information). The structural integrity, even under high-rate condition, could be due to the presence of amorphous carbon, although other factors such as dimension, cycling rate, anode fabrication method, etc. may affect this transformation. Structural integrity of nanowires is beneficial for excellent capacity performance and cyclability.^16^ Amorphous carbon coating in Ge nanowires can also positively influence specific capacity and capacity retention by drastically enhancing the reaction kinetics during cycles by promoting electron transport, increasing electrical contact points, as well as providing more paths for charge carrier transfer.^61^ This was previously confirmed for active Si anode materials with the demonstration of fast charging process and structural integrity for amorphous carbon-coated nanowires compared to nanowire without any coating.^61,62^ Significantly, we have also observed an increase in the specific capacity of C-Ge nanowires with the increase in the concentration of DPG and growth temperature (Figure S9 in the Supporting Information). This could be related to the formation of carbonaceous matrix from the polymerization of the Ge precursor, i.e., DPG, with different concentrations and at different temperatures. The specific capacity also significantly increased for C-Ge nanowires compared to our previously reported CVD-grown Ge and GeSn nanowires without any carbonaceous surroundings.^14,63^

The specific charge values obtained for our C-Ge nanowire anodes are higher than or comparable to the previously reported results for Ge nanowire- and C-Ge nanowire-based anodes, as presented in Table S1 (Ge nanowire-based anodes) and Table S2 (C-Ge nanowire-based anodes) in the Supporting Information. The C-Ge nanowire-based anode fabricated in this work shows the longest cycling stability up to 500 cycles together with the highest reversible capacity displayed at a 0.2 C cycling rate. Ge nanowires and C-Ge nanowires for Li-ion battery applications are typically grown on substrates (directly on metal or on a semiconducting substrate) via chemical vapor deposition through the use of catalytic seed and/or with high-boiling-point solvents in a reflux setup.^21,64^ Our nanowires did not require any external seeding or the use of sophisticated precursors, solvents, and setup, and can be directly grown on metal Ti substrates. Some commonly used metal seeds, such as Au, form irreversible alloys with Li during cycling and result in lower capacity values due to electrochemically inactive seeds.^65^ Furthermore, it is worth noting that the impressive large specific charges and stable capacity retention of the C-Ge nanowires were achieved in the absence of any binders of conductive additives. The electrochemical performance with a very high specific charge for the nanowires demonstrates their potential as advanced anode material for Li-ion batteries. As the main focus of this article is to develop a simplistic growth method, i.e., a supercritical fluid-based batch method for functional carbonaceous materials (C-Ge nanowires), we delegate a more detailed exploration of LIB performance of C-Ge nanowires, such as study on rate capability, cyclic voltammetry, and cycling at other current rates, to a later study.

## 3 Conclusions

In summary, an alternative synthetic method for the growth of Li-ion battery relevant C-Ge composite nanowires has been developed. In situ polymerization of a simple germanium precursor at moderate growth temperatures in a supercritical batch setup leads to the growth of highly functional C-Ge nanocomposite anode material for Li-ion batteries. The Ge nanowire growth protocol postulated here could potentially be implemented for other carbonaceous nanomaterials such as Si, SiGe, etc. via in situ polymerization of the reaction byproducts in a supercritical atmosphere. The C-Ge nanowires demonstrated exceptional performance as Li-ion battery anodes, capable of delivering impressively high specific charge values of >1200 mAh g^–1^ after 500 cycles at 0.2 C, with very low capacity decay. To our knowledge, the demonstrated electrochemical results represent some of the best electrochemical performances ever reported for Ge nanowires, therefore demonstrating the advantages of direct nanowire growth within a carbon-based matrix.

## Supporting Information Available

Detailed experimental methods, SEM images of all of the C-Ge nanowires at different growth conditions, XPS spectra of C-Ge nanowires, diameter distribution of all of the C-Ge nanowires at different growth conditions, TEM and SEM images of initial nanoparticle formation and regular seed at the tip of C-Ge nanowires, GC spectra from the analysis of the residual liquid present in the reactor after the reaction and simulated reaction densities from in situ pressure and temperatures measurements, SEM images and XRD pattern of the C-Ge nanowires grown on Ti foil, SEM of C-Ge nanowire after cycling, table of previously reported cycling performance of Ge nanostructures and carbonaceous Ge nanostructures as anode electrodes (PDF)
