# Supplementary material for: One-Step Grown Carbonaceous Germanium Nanowires and Their Application as Highly Efficient Lithium-Ion Battery Anodes
Source: ACS Appl Energy Mater. 2022 Jan 19;5(2):1922–32. doi: 10.1021/acsaem.1c03404 (PMC8889535; doi:10.1021/acsaem.1c03404)
Supplement: Supplementary file 1 — ae1c03404_si_001.pdf [file ae1c03404_si_001.pdf]

Supporting Information

One-step Grown Carbonaceous Germanium Nanowires and their Application as Highly-efficient Lithium-ion Battery Anodes

Adrià Garcia, Subhajit Biswas, David McNulty, Ahin Roy, Sreyan Raha, Sigita Trabesinger, Valeria Nicolosi, Achintya Singha and Justin D. Holmes*

[1]School of Chemistry & Tyndall National Institute, University College Cork, Cork, T12 YN60, Ireland. [2]AMBER Centre, Environmental Research Institute, University College Cork, Cork, T23 XE10, Ireland. [3]Battery Electrodes and Cells, Electrochemistry Laboratory, Paul Scherrer Institute, Forschungsstrasse 111, 5232 Villigen PSI, Switzerland. [4]Bernal Institute & Chemical Sciences Department, University of Limerick. [5]School of Chemistry and CRANN, AMBER Centre, Trinity College Dublin, Dublin 2, Ireland. [6]Department of Physics, Bose Institute, 93/1, A.P.C Road, Kolkata, 700009, India.

*Author to whom correspondence should be addressed: Tel: +353 (0)21 4905143; E-mail: s.biswas@ucc.ie

Experimental

Nanowire Synthesis. Anhydrous toluene 99.8% was purchased from Sigma-Aldrich Co and diphenylgermane (DPG) 95 % was purchased from Fluorochem. These were stored and used under inert conditions (O_2 < 0.1 ppm, H_2O < 0.5 ppm) inside a nitrogen-filled glovebox.

Ge-nanowire synthesis was carried out in a single stainless steel reaction cell (5 ml). Prior to synthesis, the reaction cell and connectors were dried under vacuum at 125 ºC for 12 hr. Reactions were performed at temperatures between 380 to 490 ºC on Si (100) substrates (native oxide present) of 0.5 × 1.5 cm dimension. The reaction temperature was monitored by a thermocouple connected to the reaction vessel and the pressure was monitored via a pressure gauge, connected to one end of the reaction cell. In general, toluene/diphenylgermane solution (3 ml) was added to the reaction cell (5 ml) and the cell was heated to the desired temperature in a tube furnace for 15–150 min. The DPG concentration was varied between 40 and 120 mᴍ. The filling volume of the reactant solution; *i.e.* filling fraction, was 20, 40, 60 and 80 % of the total reactor's volume (5 ml). The reaction cell was cooled to room temperature after the reaction and disassembled to access the growth substrate. Growth substrates were washed with dry toluene and dried under N_2 flow for further characterization. For electrochemical testing, the synthesis procedure was repeated with Ti foil instead of Si wafers as substrates, with 40 mᴍ of DPG at an optimal growth temperature of 440 °C.

Structural and chemical characterization. Samples were imaged using an FEI Quanta FEG 650 scanning electron microscope (SEM) operated at 15 kV. High-Resolution Transmission Electron Microscopy (HRTEM) and High-Resolution Scanning Transmission Electron Microscopy (HRSTEM) imaging were performed on a JEOL 2100 electron microscope operated at 200 kV and an FEI Titan electron microscope, operating at 300 kV. High-angle annular-dark-field scanning-transmission-electron-microscopy (HAADF-STEM) was performed on the FEI Titan electron microscope operated at 300 kV. XPS spectra were acquired on an Oxford Applied Research Escabase XPS system, equipped with a CLASS VM 100-mm-mean-radius hemispherical electron-energy-analyzer with a five-channel detector arrangement in an analysis chamber with a base pressure of 10×10^{-10} mbar. Raman scattering analysis was performed using a Lab RAM HR (Jobin Yvon) spectrometer equipped with a 488

nm laser source and a CCD detector. The laser was focused on the sample using a 100X objective. The laser power was maintained at 0.18 mW throughout the measurement and the data acquisition time was 50 s. Raman scattering analysis was performed on the nanowires using a low power (0.18 mW); to avoid laser-induced heating which can cause structural changes in the nanowires and a red-shift in the Ge-Ge phonon vibration. The Raman spectra for the nanowires was recorded over a wavenumber range between 100 - 2000 cm−1. The crystal structure of the product was confirmed by X-ray diffraction (XRD) using a Philips X'pert Pro MPD, equipped with a Panalytical Empyrean Cu X-ray tube and a Philips X'celerator detector. Gas chromatography-mass spectroscopy was performed on an Agilent 6890N GC equipped with a 5973 inert Mass Selective Detector (Agilent Technologies, Waldbronn, Germany). A capillary column HP-5MS [(5%-phenyl)-methylpolysiloxane] Agilent J & W GC column, 30 m, 0.25 mm i.d., coating thickness 0.25 μm was used to separate sample products. Fourier-transform infrared spectroscopy (FTIR) spectra were recorded on an infrared spectrometer (IR 660, Varian) in the range of 400 to 4000 cm^{-1}.

Electrochemical testing. The electrochemical properties of C-Ge nanowires were investigated in a half-cell configuration against a metallic Li counter electrode (diameter: 13 mm, thickness: 750 μm, Alfa Aesar) in a two-electrode, stainless steel coin-type cell. The cell was filled with the electrolyte (200 μl), consisting of a 1 M solution of lithium hexafluorophosphate salt ($LiPF_6$) in a 1:1 (v/v) mixture of ethylene carbonate and dimethyl carbonate with 3 wt.% vinylene carbonate as an SEI-forming additive. A Celgard 2400 (diameter: 17 mm, thickness: 25 μm) was used as a separator in all electrochemical tests. The mass loading for all C-Ge NW samples was ~0.1 mg/cm^2. A Mettler Toledo XP2U ultra microbalance was used to determine the mass of C-Ge NW material on the Ti foil substrates. No conductive additives or binders were added to the electrodes. Galvanostatic cycling was performed using an Arbin Battery Tester at 0.2 C (1 C = 1384 mA/g) in a potential window of 1.5 – 0.01 V (vs Li/Li^+).

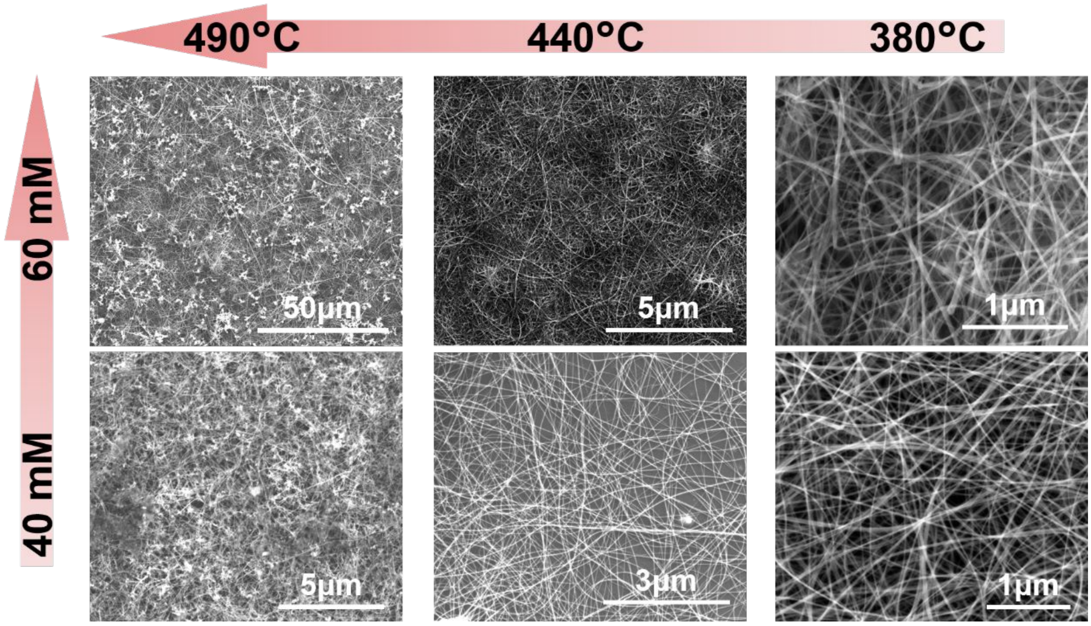

Figure S1. Representative SEM images of nanowires obtained at different temperatures and DPG concentrations.

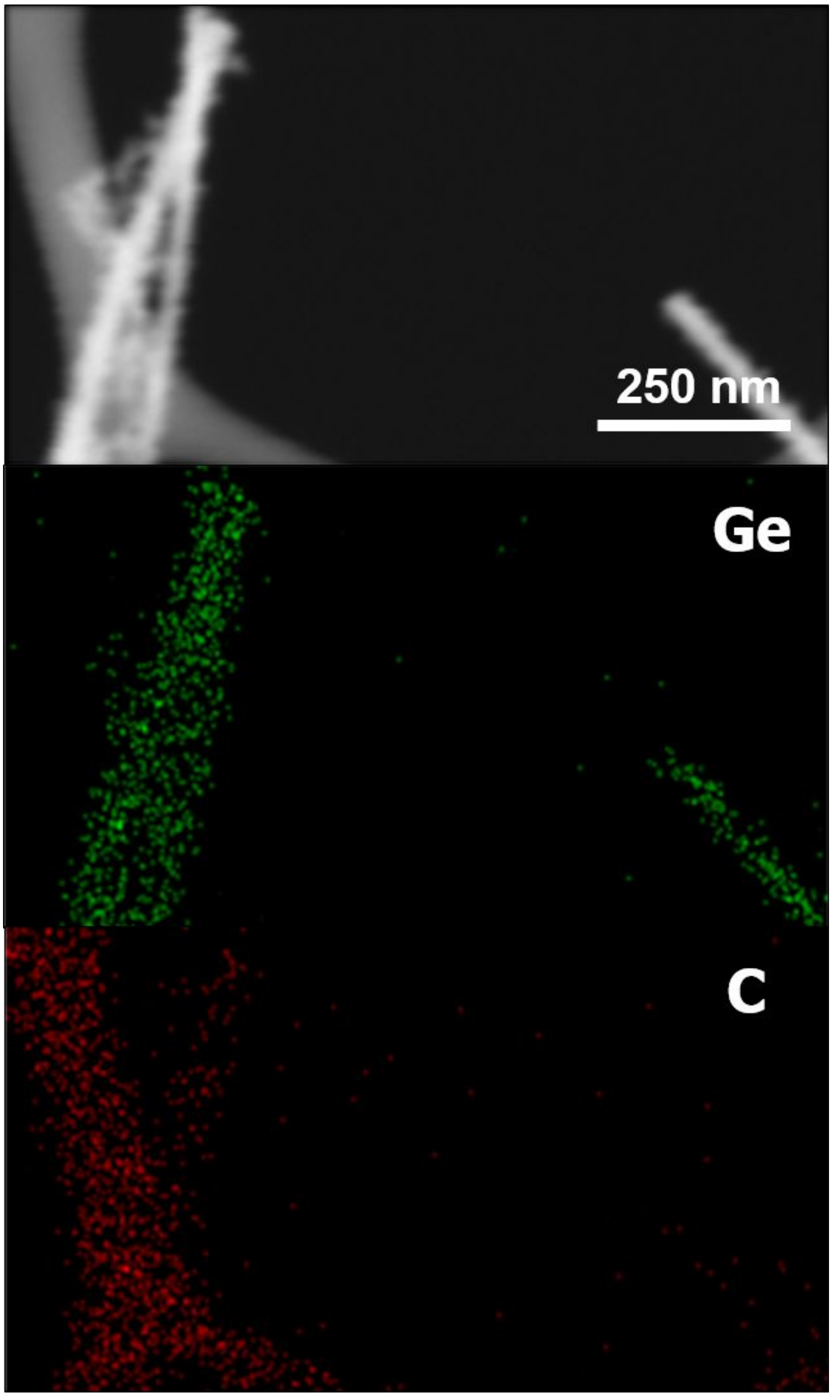

Figure S2. Dark-field STEM image and corresponding Ge and C map of C-Ge nanowires grown at 440 °C with 60 mM DPG concentration.

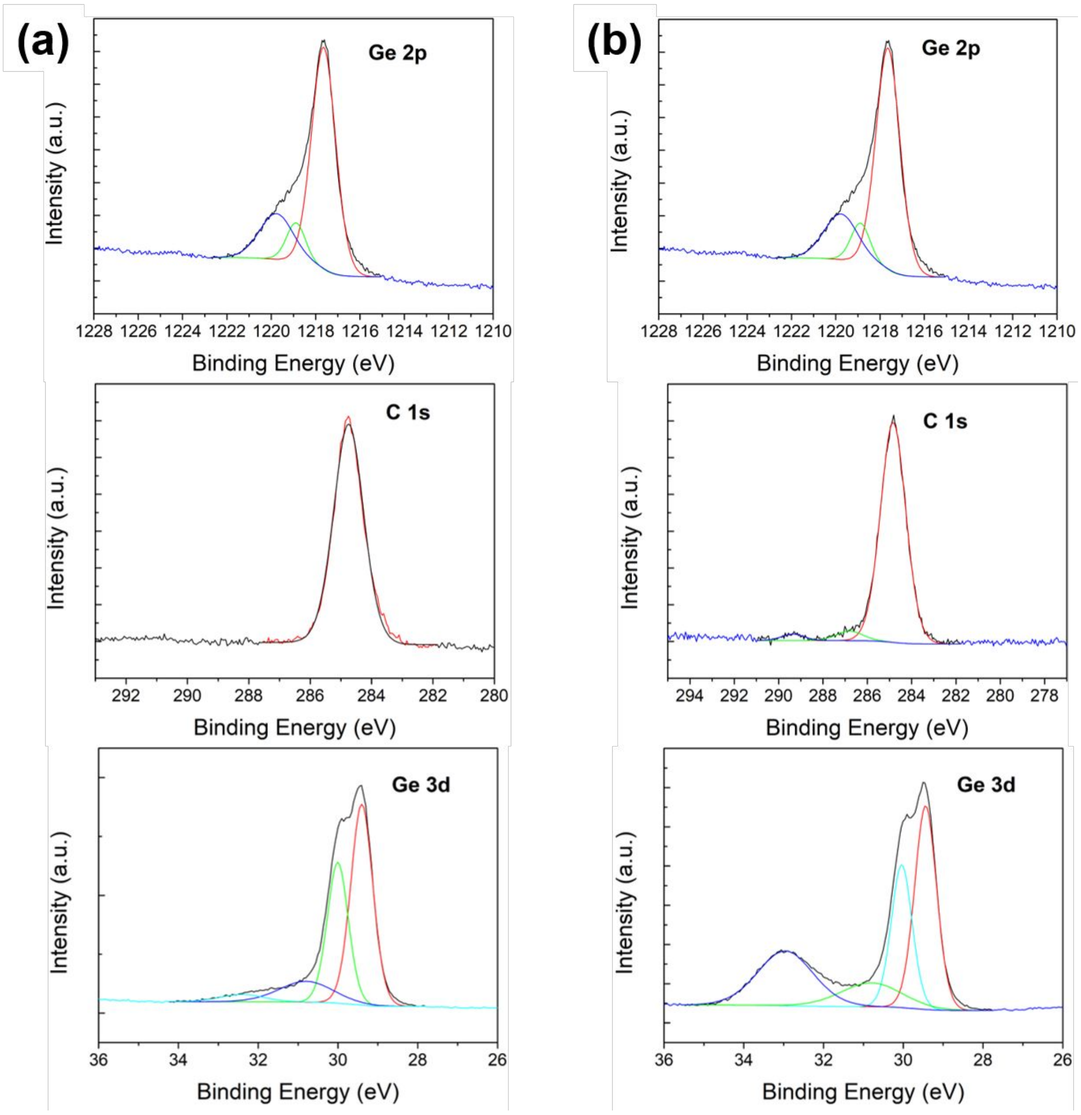

Figure S3. XPS spectra of Ge 3d, Ge 2p and C 1s sections from an (a) 60 mM solution at 380 °C, and (b) 60 mM solution at 440 °C.

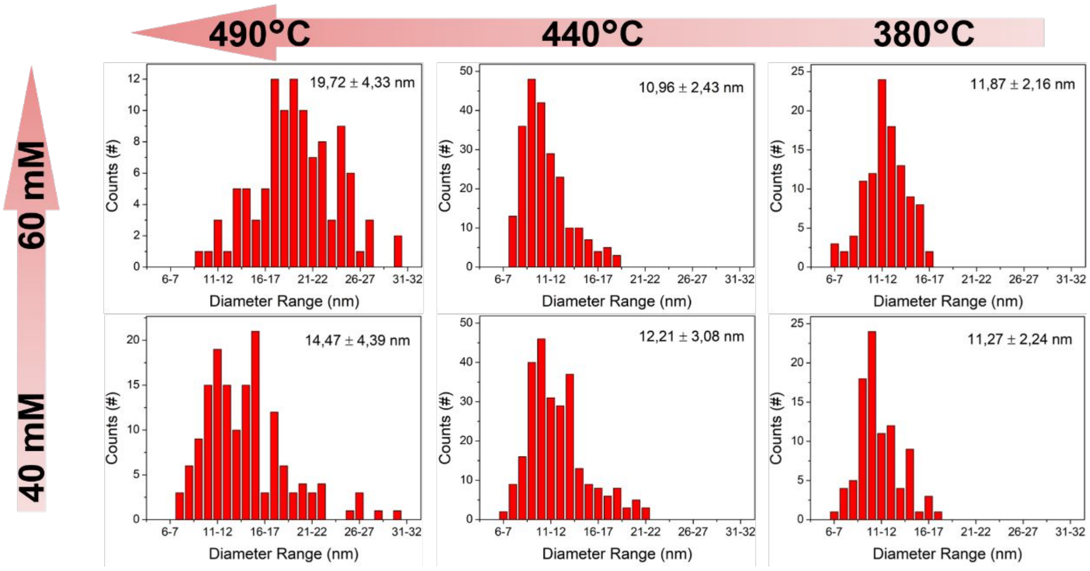

Figure S4. Diameter distribution of nanowires obtained at different temperatures and with different DPG concentrations. The average diameter is noted along with its standard deviation.

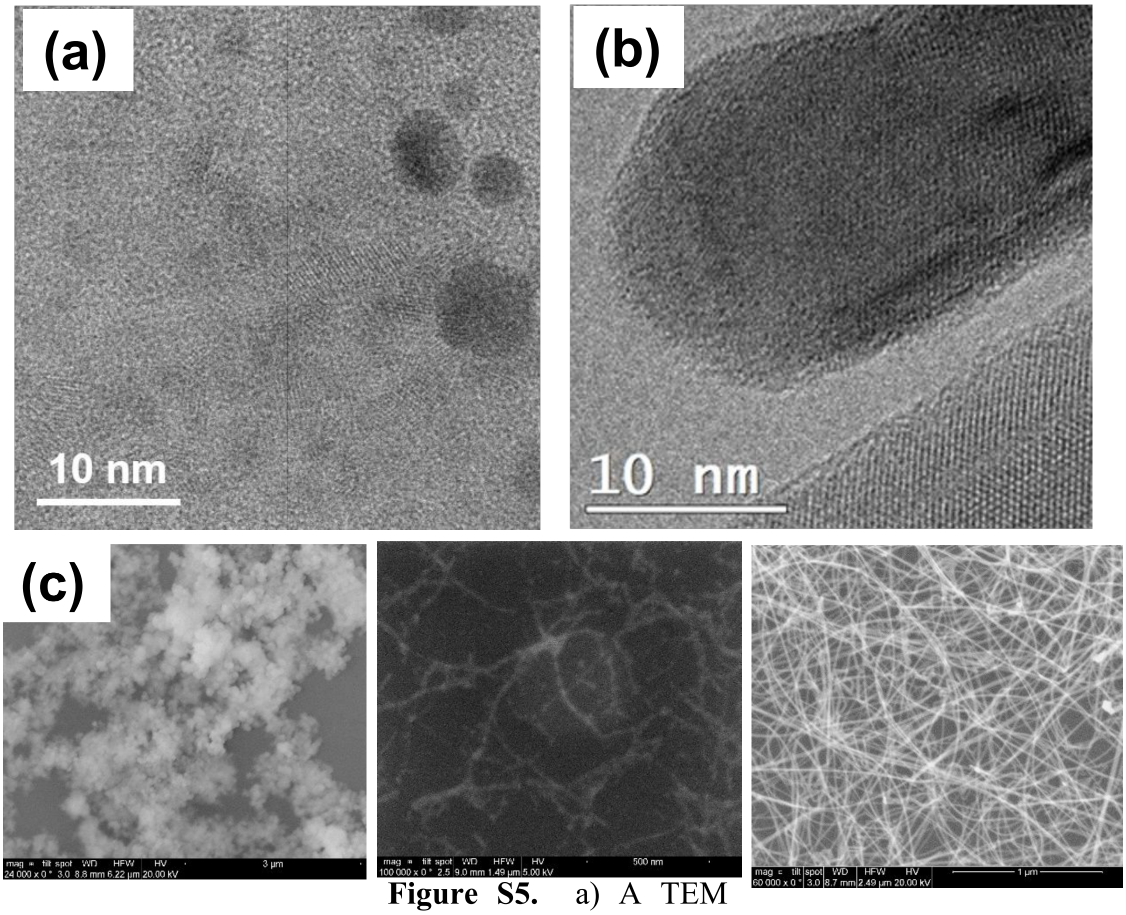

Figure S5. a) A TEM image of initial nanoparticle formation which acts as nanowire seed. Sample obtained with a reaction at a temperature of 440 °C and a 20% loading volume of the reactor. Initial nanoaprticle size matches very well with the nanowire diameter.TEM in (b) show a regular seed like feature at the tip of a carbonaceous-Ge nanowire. Part (c) show different stages of nanowire growth: nanoparticle formation, intiation of nanowire growth and fully grown nanowire.

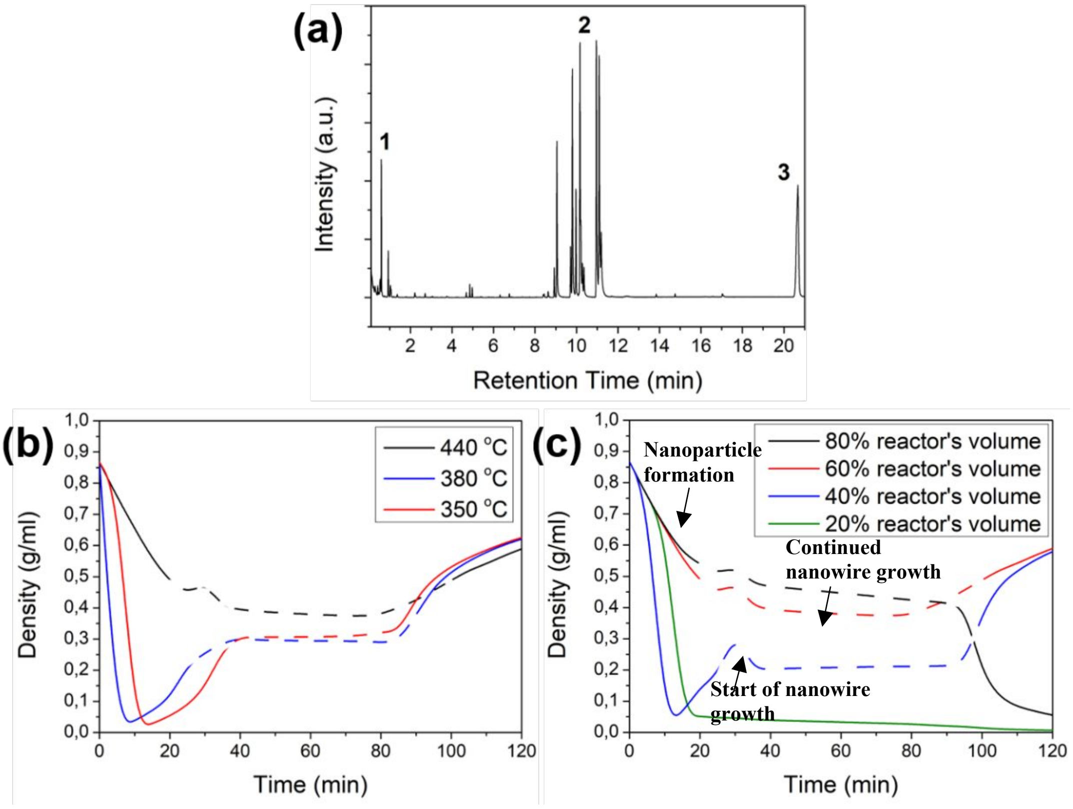

Figure S6. (a) Gas-chromatography spectra from the analysis of the residual liquid present in the reactor after the reaction. The spectra presents three zones such as: (1) Toluene-like structures and derivatives (*i.e.* ethylciclohexane, ethylbenzene and *o*-xylene), (2) diphenylmethane and derivatives (*i.e.* 2,3'-dimethyl-1,1'-byphenyl, bybenzyl or 1-methyl(4-phenylmethyl)benzene) and (3) tetraphenylgermane (QPG). (b) and (c) display calculated densities[1] of the solvent from pressure and temperatures measurement. Dashed areas of the curves indicate that supercritical state. (b) Density of the toluene at different temperatures with 60% reactor's volume loading. (c) Density of the toluene with different volume loading into the reactor at 440 °C. All the measurements are independent of the Ge precursor concentration.

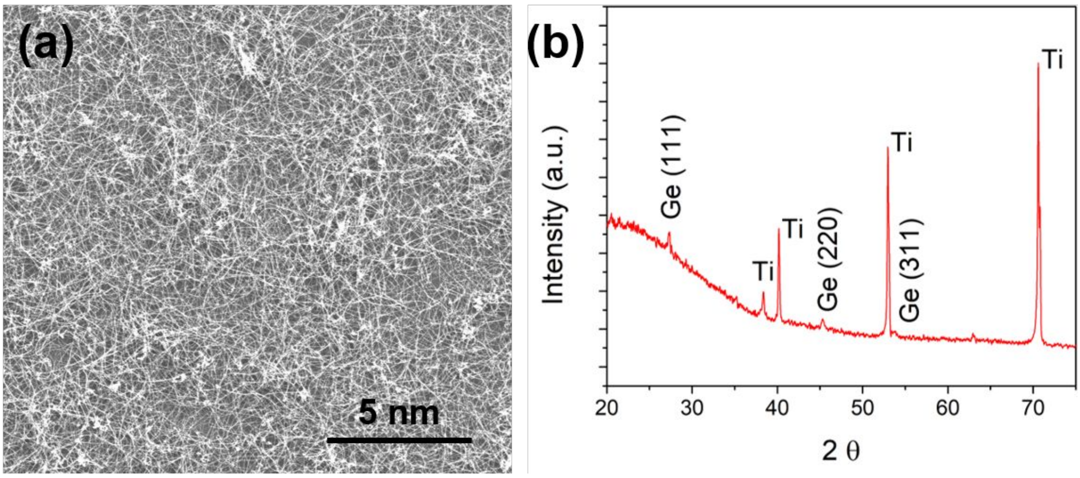

Figure S7. (a) SEM micrograph of C-Ge nanowires grown over Ti substrate at 440 from 40 mM. (b) XRD pattern of a nanowire sample showing a diamond cubic structure of Ge crystals over a Ti substrate.

.

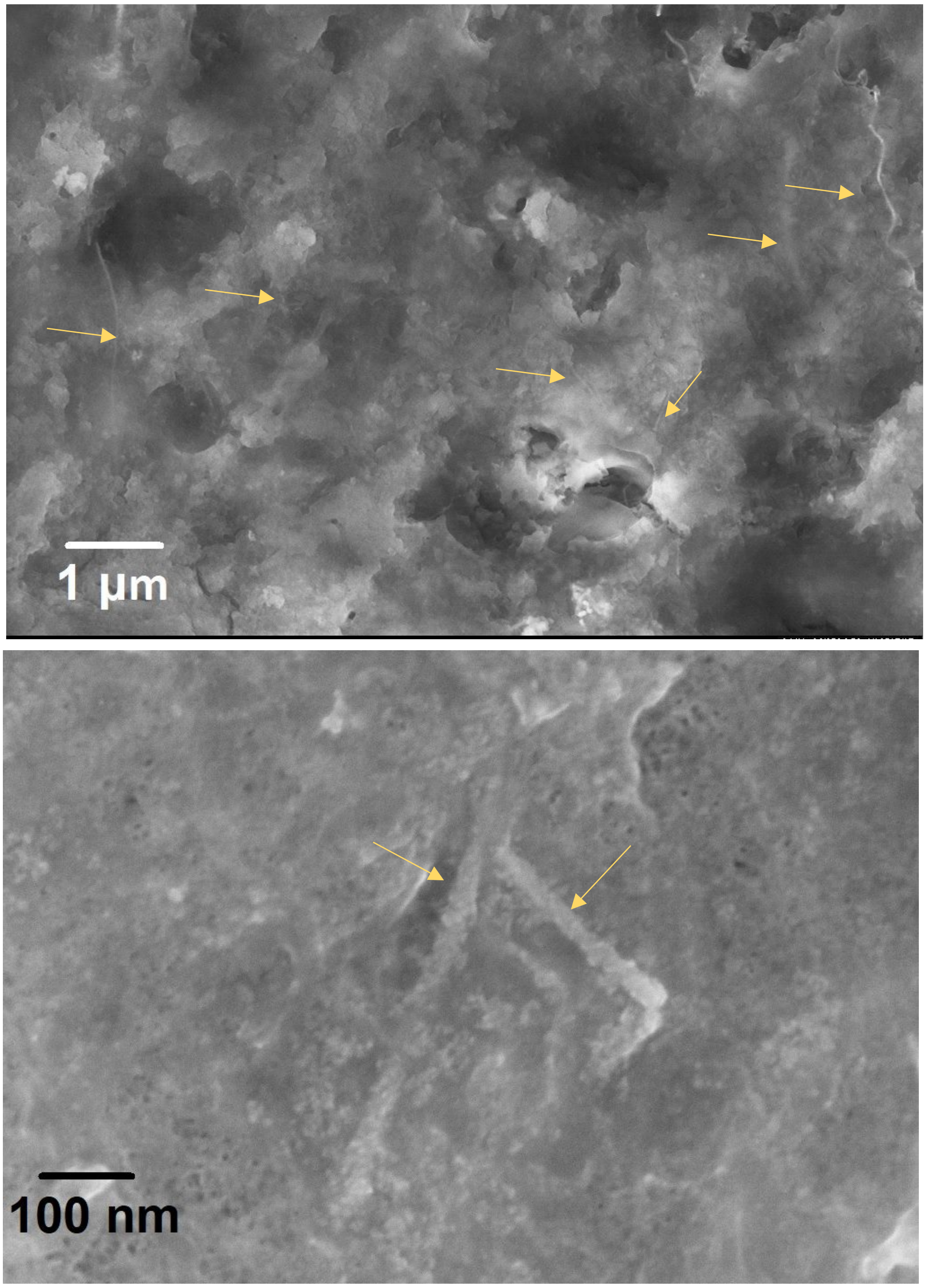

Figure S8. (a) SEM micrograph of C-Ge nanowire sample after 500 cycles. (a) A general overview of the sample showing the formation of mesh like structure and retention of 1d morphology in many instances (indicated by arrow). (b) A magnified view of the mesh and 1d morphology after electrochemical cycles

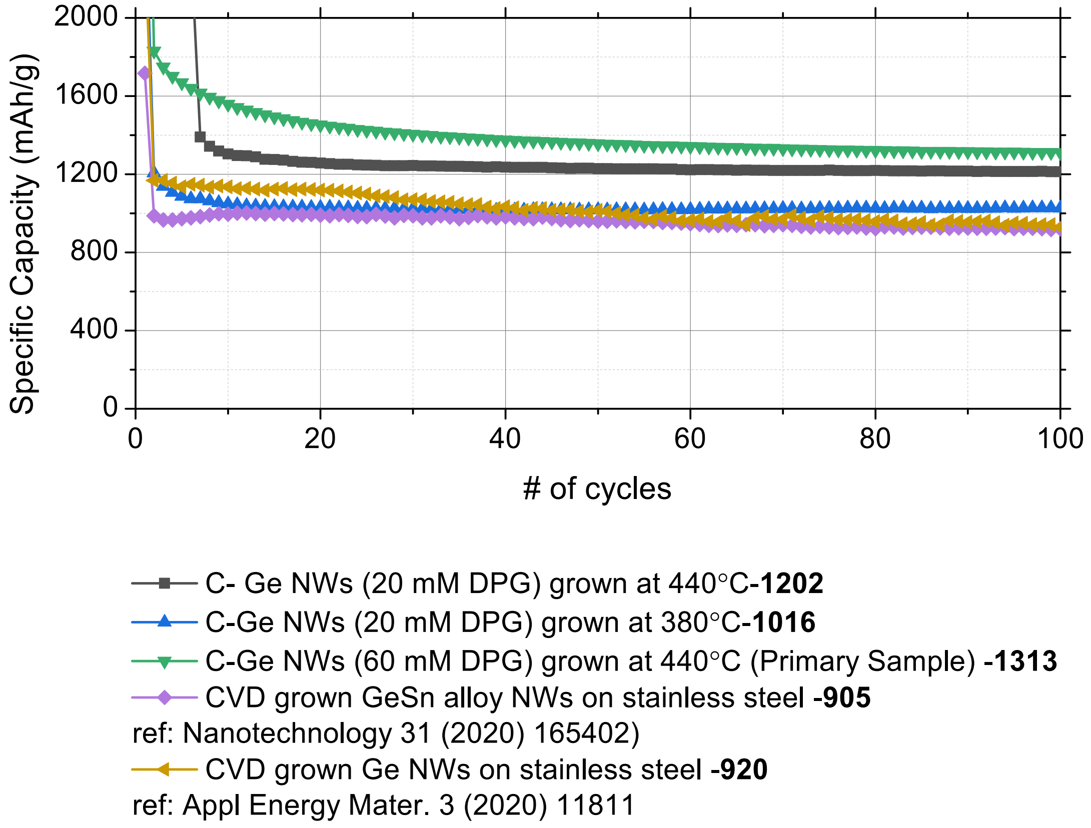

Figure S9. Comparison of the specific charge values C-Ge nanowires and CVD-grown Ge nanowires obtained at different conditions over 100 cycles. Corresponding specific charge values for the samples after 100 cycles are given bold font in the figure legend.

Table S1. Cycling performance of Ge nanostructures previously reported as anode electrodes.

Ge nanowires and nanotubes	Preparation method	Electrochemical performance	Cycles tested	Electrode preparation	Ref.
**Ge nanowires and branched nanowires**	SLS and VLS	1072 mAh g^{-1} at 0.5 C	50 cycles	As-synthesized (SS)	[2]
**Ge nanowire cluster arrays**	Electrodeposition	~875 mAh g^{-1} at 0.2 C	100 cycles	As-synthesized (Ni)	[3]
**Ge/inorganic nanowire bilayer mesh**	supercritical-fluid liquid solid growth	1092 mAh g^{-1} at 0.1 C	100 cycles	Conductive mixture	[4]
**Ge nanowires**	Vapor-liquid-solid method	~1000 mAh g^{-1} at 0.5 C	100 cycles	As-synthesized (SS)	[5]
**Ge nanowires**	CVD	1408 mAh g^{-1} at 0.1 C	100 cycles	As-synthesized (SS)	[6]
**Ge nanowires**	Thermolytic decomposition	~1150 mAh g^{-1} at 1 C	100 cycles	As-synthesized (SS)	[7]
**Ge nanowires**	Electrodeposition	~1200 mAh g^{-1} at 0.1 C	200 cycles	As-synthesized (Ni)	[8]
**Ge nanotube arrays**	Ionic liquid electrodeposition	~1000 mAh g^{-1} at 0.2 C	250 cycles	As-synthesized (Cu)	[9]
**Nanoporous Ge nanofibers**	CVD	~1000 mAh g^{-1} at 2 C	300 cycles	Conductive mixture	[10]

Table S2. Cycling performance of carbonaceous Ge nanostructures previously reported as anode electrodes.

Carbonaceous Ge nanowires and nanotubes	Preparation method	Electrochemical performance	Cycles tested	Carbon content	Electrode preparation	Ref.
**Graphene on a-Ge nanowires**	CVD	1210 mAh g^{-1} at 0.5 C	200 cycles	≤ 4 graphene layers	Conductive mixture	[11]
**Alkanethiol-passivated Ge nanowires**	Dodecanethiol monolayer passivation	1130 mAh g^{-1} at 0.1 C	100 cycles	~3 wt.%	Conductive mixture	[12]
**Ge/C nanowires**	Pyrolysis Ge/ ethylenediamine nanowires	1200 mAh g^{-1} at 0.2 C	50 cycles	~4 wt.%	Conductive mixture	[13]
**Ge@C nanocables**	Physical vapor deposition	1086 mAh g^{-1} at 0.5 C	200 cycles	10 wt.%	Conductive mixture	[14]
**Ge nanowires on graphite nanofibers**	CVD	~1200 mAh g^{-1} at 0.1 C	30 cycles	~18 wt.%	Conductive mixture	[15]
**Ge nanowires with carbon nanofibers coating**	Vapor-liquid-solid method	~1520 mAh g^{-1} at 0.1 C	100 cycles	50 wt.%	As-synthesized	[16]
**Ge nanowire-in-Graphite Tubes**	CVD	1241 mAh g^{-1} at 0.45 C	100 cycles	Undeter-mined	Conductive mixture	[17]
**Carboneums Ge nanowire**	Solvothermal-like Supercritical-fluid synthesis	1225 mAh g^{-1} at 0.2 C	500 cycles	5-10 wt.%	As-synthesized	This work

References

(1) NIST Chemistry WebBook https://webbook.nist.gov/chemistry/ (accessed 2021 -05 -26).

(2) Flynn, G.; Palaniappan, K.; Sheehan, M.; Kennedy, T.; Ryan, K. M. Solution Synthesis of Lead Seeded Germanium Nanowires and Branched Nanowire Networks and Their Application as Li-Ion Battery Anodes. *Nanotechnology* **2017**, *28*, 255603.

(3) Chi, C.; Hao, J.; Liu, X.; Ma, X.; Yang, Y.; Liu, X.; Endres, F.; Zhao, J.; Li, Y. UV-Assisted, Template-Free Electrodeposition of Germanium Nanowire Cluster Arrays from an Ionic Liquid for Anodes in Lithium-Ion Batteries. *New J. Chem.* **2017**, *41*, 15210–15215.

(4) Chang, W. C.; Kao, T. L.; Lin, Y.; Tuan, H. Y. A Flexible All Inorganic Nanowire Bilayer Mesh as a High-Performance Lithium-Ion Battery Anode. *J. Mater. Chem. A* **2017**, *5*, 22662–22671.

(5) Kennedy, T.; Mullane, E.; Geaney, H.; Osiak, M.; O'Dwyer, C.; Ryan, K. M. High-Performance Germanium Nanowire-Based Lithium-Ion Battery Anodes Extending over 1000 Cycles through in Situ Formation of a Continuous Porous Network. *Nano Lett.* **2014**, *14*, 716–723.

(6) Farbod, B.; Cui, K.; Kupsta, M.; Kalisvaart, W. P.; Memarzadeh, E.; Kohandehghan, A.; Zahiri, B.; Mitlin, D. Array Geometry Dictates Electrochemical Performance of Ge Nanowire Lithium Ion Battery Anodes. *J. Mater. Chem. A* **2014**, *2*, 16770–16785.

(7) Mullane, E.; Kennedy, T.; Geaney, H.; Ryan, K. M. A Rapid, Solvent-Free Protocol for the Synthesis of Germanium Nanowire Lithium-Ion Anodes with a Long Cycle Life and High Rate Capability. *ACS Appl. Mater. Interfaces* **2014**, *6*, 18800–18807.

(8) Hao, J.; Yang, Y.; Zhao, J.; Liu, X.; Endres, F.; Chi, C.; Wang, B.; Liu, X.; Li, Y. Ionic Liquid Electrodeposition of Strain-Released Germanium Nanowires as Stable Anodes for Lithium Ion Batteries. *Nanoscale* **2017**, *9*, 8481–8488.

(9) Liu, X.; Hao, J.; Liu, X.; Chi, C.; Li, N.; Endres, F.; Zhang, Y.; Li, Y.; Zhao, J. Preparation of Ge Nanotube Arrays from an Ionic Liquid for Lithium Ion Battery Anodes with Improved Cycling Stability. *Chem. Commun.* **2015**, *51*, 2064–2067.

(10) Kim, C.; Song, G.; Luo, L.; Cheong, J. Y.; Cho, S.-H.; Kwon, D.; Choi, S.; Jung, J.-W.; Wang, C.-M.; Kim, I.-D.; Park, S. Stress-Tolerant Nanoporous Germanium Nanofibers for Long Cycle Life Lithium Storage with High Structural Stability. **2018**.

(11) Kim, H.; Son, Y.; Park, C.; Cho, J.; Choi, H. C. Catalyst-Free Direct Growth of a Single to a Few Layers of Graphene on a Germanium Nanowire for the Anode Material of a Lithium Battery. *Angew. Chemie - Int. Ed.* **2013**, *52*, 5997–6001.

(12) Yuan, F. W.; Yang, H. J.; Tuan, H. Y. Alkanethiol-Passivated Ge Nanowires as High-Performance Anode Materials for Lithium-Ion Batteries: The Role of Chemical Surface Functionalization. *ACS Nano* **2012**, *6*, 9932–9942.

(13) Liu, J.; Song, K.; Zhu, C.; Chen, C. C.; Van Aken, P. A.; Maier, J.; Yu, Y. Ge/C Nanowires as High-Capacity and Long-Life Anode Materials for Li-Ion Batteries. *ACS Nano* **2014**, *8*, 7051–7059.

(14) Yue, G. H.; Zhang, X. Q.; Zhao, Y. C.; Xie, Q. S.; Zhang, X. X.; Peng, D. L. High Performance of Ge@C Nanocables as the Anode for Lithium Ion Batteries. *RSC Adv.* **2014**, *4*, 21450–21455.

(15) Woo, S.-H.; Choi, S. J.; Park, J.-H.; Yoon, W.-S.; Hwang, S. W.; Whang, D. Entangled Germanium Nanowires and Graphite Nanofibers for the Anode of Lithium-Ion Batteries. *J. Electrochem. Soc.* **2013**, *160*, A112–A116.

(16) Li, W.; Li, M.; Yang, Z.; Xu, J.; Zhong, X.; Wang, J.; Zeng, L.; Liu, X.; Jiang, Y.; Wei, X.; Gu, L.; Yu, Y. Carbon-Coated Germanium Nanowires on Carbon Nanofibers as Self-Supported Electrodes for Flexible Lithium-Ion Batteries. *Small* **2015**, *11*, 2762–2767.

(17) Sun, Y.; Jin, S.; Yang, G.; Wang, J.; Wang, C. Germanium Nanowires-in-Graphite

Tubes via Self-Catalyzed Synergetic Confined Growth and Shell-Splitting Enhanced Li-Storage Performance. *ACS Nano* **2015**, *9*, 3479–3490.
